# Superior component compositions and antioxidant activity of *Volvariella volvacea* oil compared to those of *Agrocybe cylindracea* and two *Lentinula edodes* oils

**DOI:** 10.1002/fsn3.3750

**Published:** 2023-10-16

**Authors:** Jingjing Kang, Yue Yue, Shaofeng Wei, Huifang Chen, Peng Luo

**Affiliations:** ^1^ The key Laboratory of Environmental Pollution Monitoring and Disease Control, Ministry of Education, School of Public Health Guizhou Medical University Guiyang China

**Keywords:** antioxidant capacity in vitro, lipid composition, minor elements, mushroom oil

## Abstract

The biological activity of an oil not only depends on its fatty acid composition but also the lipid composition and trace components. In this paper, to select the optimal mushroom oil, the component compositions (fatty acids, lipids, polyphenols, flavones, tocopherols, and unsaponifiable matters) and antioxidant activities in vitro of four mushroom oils (*Agrocybe cylindracea*, two *Lentinula edodes*, and *Volvariella volvacea)* were investigated and compared. The results showed that the four tested oils had the same fatty acid composition in different amounts, but the lipid component, minor components, and free radical scavenging activity in the tested oils varied widely depending on the type of mushroom. Overall, *Volvariella volvacea* oil was considered superior to the other three tested oils, as it had the largest contents of polar lipids, diglycerides, polyunsaturated fatty acids (74.38%), unsaponifiable matter (319.09 mg/kg), total phenols (124.08 mg/100 g), tocopherols (139.86 mg/100 g), as well as the highest ABTS and FRAP values (349.45 and 3801.70 μmol Trolox/100 g). This finding suggests that *Volvariella volvacea* oil is a promising resource that should be further researched.

## Graphical abstract

There is a great significant difference in minor components and free radical scavenging activity among the four test mushroom oils. *Volvariella volvacea* oil was proven as the optimal mushroom oil needed for further research and development.

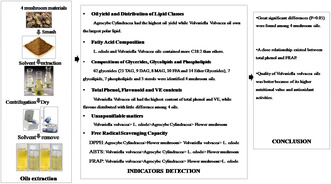

## 1 INTRODUCTION

With the increasing desire for greater sustainability and food security, more and more new resources have been developed to enrich food diversity (Rosmiza et al., 2016); thus, mushrooms have gained attention because of their high nutritional value (rich in protein, carbohydrates, edible fiber, vitamins, and phytosterols), medicinal activity (antitumor, hipocolesterolemic, antioxidant, antifungal, and antimicrobial), and industrial application (Kitzberger et al., 2007, 2009; Sugui et al., 2003). Apart from their appearance as food ingredients at dinner tables, mushrooms are also applied in cosmetics (Wu et al., 2016), health care (Lu et al., 2020), and pharmaceutical fields (Valverde et al., 2015). At present, the mushroom industry is regarded as important for rural areas to prosper because it benefits agriculture and increases farmers' income in many countries. Therefore, it is necessary to discuss any approach to enhance the added value of edible mushrooms.

Given the early studies on their chemical composition and nutritional qualities, mushrooms are labeled “high in proteins, vitamins, fibre and minerals” but “low in calories, fats, and essential fatty acids” (Agrahar‐Murugkar & Subbulakshmi, 2005; Sanmee et al., 2003). Compared with macronutrients such as polysaccharides, polypeptides, and dietary fiber, oil (or lipid), accounting for 1.75–15.5% of the dry weight of mushrooms (Hong et al., 1988), is often ignored by researchers, resulting in insufficient studies. However, oil (or lipid) is an important carrier of fat‐soluble active constituents (i.e., tocopherols, ergosterols, terpenoids, and so on), which play an important role in the function of mushrooms, as the fat‐soluble components of mushrooms have been reported to present biological activities, such as antioxidant, antitumor, and immunomodulatory activities. Therefore, to evaluate the medicinal value of mushrooms comprehensively, it is essential to study mushroom oil.

Regarding the existence of fat‐soluble active constituents, the biological activity of mushroom oil is also separately affected by these trace concomitant contents, in addition to being affected by fatty acid and lipid composition (Zheng et al., 2017). Hence, when we need to explore the potential application value of mushroom oil, it is crucial to investigate the extract composition and content of the lipids and microcomponents in mushroom oil.

Early in 2013, *L. edodes* constituted 22% of the world's total output and was affirmed as the most widely cultivated mushroom (Royse et al., 2017). However, during the initial cultivation period, *L. edodes* was only cultivated in certain regions with suitable climate, its limited production could not meet the increasing demand of people, and it was the shortage of *L. edodes* that intensified the expansion of cultivation areas. *Flower mushrooms* are the product of abnormal growth of *L. edodes*, and unsuitable planting environmental conditions (i.e., low temperature and humidity) could promote the transformation of *L. edodes* to *flower mushrooms*. In contrast to conventional *L. edodes*, which have *s*mooth pileipellis, *flower mushrooms* are produced only when the fleshy cells of *L. edodes* rupture to form chrysanthemum‐like pilei (Cao, 2000). Although *flower mushrooms* are 5–8 times more expensive than conventional *L. edodes*, they are still preferred by global consumers owing to the higher nutritional value and superior characteristics (thick pilei, tender meat, and beautiful appearance) (Liu et al., 2022). With an output accounting for 5% of the world's total production, *Volvariella volvacea* is deemed the second most widely cultivated mushroom. Similar to *L. edodes*, *Volvariella volvacea* and *Agrocybe cylindracea* have also recently been accepted by more and more consumers because of their delicious taste and high nutritive value (Jia et al., 2020). Therefore, based on their enormous output and high popularity, these four mushrooms *(Agrocybe cylindracea*, *L. edodes*, *flower mushroom*, and *Volvariella volvacea*) occupy a pivotal position in the mushroom industry.

In this study, oils of *Agrocybe cylindracea*, conventional *L. edodes*, *flower mushroom*, and *Volvariella volvacea* were extracted to investigate their exact nutritional composition and free radical scavenging ability. By comparing all tested indicators, the oil with the highest development value was selected, providing theoretical data for the future application of mushroom oil.

## 2 MATERIALS AND METHODS

### 2.1 Materials

Dried mushrooms (*Agrocybe cylindracea*, conventional *L. edodes*, *flower mushroom*, and *Volvariella volvacea*) were provided by Xi'an ZHONGKEDA Biotechnology Co., Ltd (a company selling plant extracts and animal extract production specifically). Standards of α‐, γ‐, and δ‐tocopherols, Trolox, and cholestan‐3‐ol (purity >98%) were purchased from Toronto Research Chemicals Inc (Shanghai, China). The antioxidant reagents 1,1‐diphenyl‐2‐picrylhydrazyl (DPPH), 2,2′‐azinobis‐(3‐ethylbenzthiazoline‐6‐sulfonate) (ABTS), and tripyridyltriazine (TPTZ) were provided by Sigma‐Aldrich Chemical Co., Ltd. (Shanghai, China), while the other reagents and solvents were offered by Shanghai Maclin Biochemical Technology Co., Ltd.

### 2.2 Mushroom oil preparation

The solvent extraction method was used to prepare the mushroom oil. Specifically, the mushroom sample was washed, dried, and smashed through an 80‐mesh sieve to extract oil using petroleum ether (boiling range, 60–90°C) at a ratio of 4:1 (V/W), after 4 h of oil extraction at room temperature, and the mushroom oil was mainly distributed into the supernatant, which needed to be isolated from the mushroom sediment by centrifugation. After removing the solvent from the supernatant, mushroom oil was obtained and stored at −20°C for analysis.

### 2.3 Oil Characteristics

#### 2.3.1 Oil yield

Oil yield (%) was calculated by the mass ratio of oil to the mushroom sample after oil extraction.

#### 2.3.2 Lipid separation

According to Heinzelmann et al. (2014), a silica gel column was applied to separate the polar lipids (phospholipids and glycolipids) from the neutral lipids (glycerides) of mushroom oil, namely the polar lipids and neutral lipids of mushroom oil were separated by a silica gel column (30 × 400 mm) containing approximately 35 g active silica gel. After adding 25 mg/mL oil chloroform solution into the column, chloroform, acetone, and methanol were used to wash the column in turn to obtain fractions of glycerides, glycolipids, and phospholipids, respectively.

#### 2.3.3 Fatty acid composition

GC–MS was applied to determine the fatty acid composition of the mushroom oil and the procedures were performed according to Kang et al. (2019) with some modifications. In brief, a 20 mg oil sample and 3 mL sodium methoxide solution (0.5 M) were mixed together to enable saponification for 30 min at 70°C, and then, 1 mL boron trifluoride diethyl ether solution was added into the mixture for water bath heating at 80°C for 30 min, which enhanced the production of the fatty acid methyl ester. After the alkaline reaction liquid cooled to room temperature, 3 mL of heptane was added to extract the fatty acid methyl ester from mixture. After the evaporation of solvent, the dried oil extract was re‐dissolved, filtered (13 mm, 0.22 μm), and injected into the GC–MS for fatty acid analysis.

GC–MS for determining the fatty acid composition was equipped with a DB‐5 column (30 m × 0.25 mm × 0.25 μm), and the temperatures of the injector and detector were both set at 290°C. At a sample loading of 1.0 μL, the sample was analyzed at a split ratio of 100/1 and an oven temperature program containing 180°C (2 min), 10°C/min to 230°C (1 min), 3°C/min to 236°C (1 min), 6°C/min to 260°C (2 min), and 5°C/min to 270°C (2 min). Temperatures of ion source and transmission line were 250°C and 280°C, respectively, for EI Ionization mode at a scan range of 100–350 amu.

#### 2.3.4 Lipid composition

A Thermo Scientific UltiMate 3000 HPLC equipped with an ACQUITY UPLC BEH C18 column (100 mm × 2.1 mm, 1.8 μm, Waters, UK) and a high‐resolution tandem mass spectrometer Q Exactive (Thermo Scientific) was used to analyze triacylglycerol composition of the lipid composition of the mushroom oils. After setting the injection volume, column oven temperature, and flow rate at 4 μL, 35°C, and 0.4 mL/min, respectively, the oil sample was tested by a gradient elution program containing mobile phase A (water, 0.1% formic acid) and mobile phase B (acetonitrile, 0.1% formic acid). The exact elution conditions were as follows: 0–0.5 min, 5% B; 0.5–7 min, 5% to 100% B; 7–8 min, 100% B; 8–8.1 min, 100% to 5% B; and 8.1–10 min, 5% B.

The mass spectrometer was operated in both positive and negative ion modes. At maximum injection times of 100 ms and 80 ms, precursor spectra (70–1050 m/z) and fragment spectra were collected at resolutions of 70,000 and 17,500, respectively, to hit AGC targets of 3e^6^ (precursor) and 1e^5^ (fragment). DDA mode was brought to acquire data in the top three configurations, and the stability of the UPLC‐MS was evaluated by a quality control sample after every 10 tested samples.

#### 2.3.5 Total phenol, flavone, and tocopherol content

0.5 mL of 90% aqueous methanol was used to extract total phenol and flavone from 50 mg of mushroom oil successively three times, and the methanol supernatant was collected to evaporate to dryness. After redissolving the dryness into 0.5 mL of methanol, the mixed methanol solution (i.e., the oil methanol extract) was filtered through a hydrophilic syringe membrane filter (0.45 μm) for analysis (Grajzer et al., 2020). The spectrophotometric determination of total phenol and total flavones was carried out according to Choo et al. (2007) and Siger et al. (2008), respectively.

A 0.1 g/mL oil n‐hexane solution was filtered for tocopherol analysis, which requires an HPLC device equipped with a silica gel column ZORBAX RX‐SIL (4.6 mm × 250 mm, 5 μm) and ultraviolet detector (Cunha et al., 2006). At a flow rate of 1 mL/min, the temperature of the column was set at 35°C, with n‐hexane/isopropanol/glacial acetic acid at 99/0.2/0.8 (V/V/V) as the only mobile phase, and 10 μL of sample solution was analyzed by a 20 min isocratic elution procedure. The tocopherol content in mushroom oil was determined by the external standard method.

#### 2.3.6 Unsaponifiable matter

After saponifying the mushroom oil by alkaline hydrolysis, the mixture containing alcoholic potassium hydroxide was extracted by n‐hexane to obtain unsaponifiable matter. To ensure full saponification and precise quantification, the oil sample (0.2 g) was mixed with 3 mL of potassium hydroxide solution (2 M) and 0.5 mL of internal standard (5α‐cholestane, 0.1 mg/mL) and heated for 30 min at 80°C. The hexane solvent was evaporated to dryness and 200 μL BSFA‐TMCS was added for silanization. Based on the internal standard method of quantitative analysis, GC–MS was applied to analyze the exact composition of the unsaponifiable matter.

#### 2.3.7 Antiradical scavenging activity

Spectrophotometry was used to detect the antiradical scavenging activity of mushroom oil, which was evaluated comprehensively by three indicators: DPPH radical scavenging activity, ABTS radical cation decolorization assay, and the reducing ability of ferric ions in this research. With Trolox methanol solutions at different concentrations as antioxidant standards, all antioxidant indicators of mushroom oil in vitro were quantified by Trolox.

Measurement of DPPH radical scavenging activity was operated according to Nafis et al. (2019) with slight modification. Equal volumes of oil methanol extract (0.2 g/mL) and DPPH‐methanol solution (0.4 mmol/L) were mixed in the dark for 30 min. With Trolox methanol solutions (10–180 μmol/L) as standards, the DPPH radical scavenging activity of the oil sample was measured by the absorbance at 517 nm.

The ABTS radical cation decolorization assay of mushroom oil was performed based on a study by Huang et al. (2021) with a slight change. A working solution containing ABTS (3.5 mmol/L) and potassium persulfate (1.3 mmol/L) was stored at room temperature for 12–16 h before use. After adjusting the absorbance at 734 nm to 0.70, the working solution (200 μL) was mixed with oil methanol extract (0.2 g/mL, 10 μL) and reacted for 20 min at 30°C, and Trolox methanol solution (200–800 μmol/L) was also used to quantify the ABTS radical cation decolorization assay of the oil sample, which was detected at 734 nm.

The reducing ability of ferric ions in mushroom oil was measured according to the modified method of Zouirech et al. (2022). Briefly, a working solution was prepared by mixing sodium acetate trihydrate acetic acid solution (0.3 mol/L), TPTZ hydrochloric acid (10 mmol/L), and FeCl_3_ solution (20 mmol/L) at ratio of 10:1:1. Similar to the operation of ABTS, the reducing ability of ferric ions in mushroom oil was calculated by the absorbance at 593 nm, after mixing 200 μL of working solution and 10 μL of oil methanol extract at 37°C for a 10 min reaction.

#### 2.3.8 Statistical analysis

All experiments were performed in triplicate, and the results were calculated as the means and standard deviations. SPSS Statistics software was applied to find differences in data between groups, and *p*‐values <.05 were regarded as statistically significant.

## 3 RESULTS AND DISCUSSIONS

### 3.1 Oil yield and distribution of lipid category

As shown in Table 1, the oil yield of the four mushrooms ranged from 1.31 to 2.98%. Similar to glycerides, polar lipids containing glycolipids and phospholipids were also regarded as the main lipid composition of mushroom oil because of the 14.96–32.55% percentage, conforming to the low lipid content (Vidović et al., 2011) and high polar lipid proportion (Hanuš et al., 2008) in most mushrooms. *Agrocybe cylindracea* had the highest oil yield (2.98%), while *Volvariella volvacea* had the lowest oil content (1.31%). Even so, *Volvariella volvacea* oil containing 29.53% glycolipids and 3.02% phospholipids was still believed to have a better emulsifying property, which was reported to be positively correlated with the percentage of polar lipids in the oil.

**TABLE 1:** Oil yield and lipid category distribution of four test mushroom oils.

%	Mean ± SD (*n* = 3)			
	*Agrocybe cylindracea*	Conventional *Lentinula edodes*	*Flower mushroom*	*Volvariella volvacea*
Oil yield	2.98 ± 0.24^a^	2.17 ± 0.11^b^	1.84 ± 0.07^c^	1.31 ± 0.02^d^
Glycerides	81.63 ± 3.50^a^	85.04 ± 3.79^a^	84.53 ± 0.96^a^	67.44 ± 5.96^b^
Glycolipids	15.84 ± 1.30^b^	12.35 ± 2.74^c^	12.92 ± 2.66^c^	29.53 ± 0.18^a^
Phospholipids	2.53 ± 0.06^b^	2.61 ± 1.04^b^	2.56 ± 1.01^b^	3.02 ± 0.67^a^

*Note*: Datas with different superscript letter mean a significant difference (*p* < .05) betwwen two samples, while that with same letter signifys no significant difference.

### 3.2 Fatty acid composition

Although the oil yield of mushrooms was low, the mushroom oils were still abundant in unsaturated fatty acids (Yilmaz et al., 2006). According to Table 2, four tested mushroom oils were identified to possess seven fatty acids, where three fatty acids containing C16:0 (12.76–34.17%), C18:1 (3.43–24.95%), and C18:2 (40.35–75.55%) occupied the dominant position. In spite of the same molecular composition, the content of fatty acids varied from different lipid fraction as well as the mushroom species. For example, glycerides are apt to possess more polyunsaturated fatty acids (PUFAs) than polar lipids, and saturated fatty acids are more easily enriched in phospholipids sections apart from *flower mushroom* oil. More specifically, *Agrocybe cylindracea* contained 66.64%, 59.63%, 40.35%, and 63.38% PUFAs in glyceride, glycolipid, phospholipid, and whole oil, respectively. Compared to the 63.38–71.56% PUFA content in most tested mushroom oils, the 74.38% PUFA content endowed *Volvariella volvacea* oil with a special activity benefit to people who have hypertension, coronary heart diseases, ulcerative colitis, rheumatoid arthritis, chronic obstructive pulmonary diseases, and Crohn's disease (Fan & Chapkin, 1998; Kavishree et al., 2008).

**TABLE 2:** Fatty acid compositions of four test mushroom oils.

Fat acid	Content (%)/mean ± SD (*n* = 3)															
	Agrocybe cylindracea				Conventional *Lentinula edodes*				Flower mushroom				Volvariella Volvacea			
	Glycerides	Glycolipids	Phospholipids	Oil	Glycerides	Glycolipids	Phospholipids	Oil	Glycerides	Glycolipids	Phospholipids	Oil	Glycerides	Glycolipids	Phospholipids	Oil
C_14:0 (Myristic)_	0.83 ± 0.22	nd	nd	0.42 ± 0.16	0.81 ± 0.03	2.70 ± 0.02	nd	0.71 ± 0.27	0.73 ± 0.19	1.15 ± 0.33	nd	0.72 ± 0.18	1.91 ± 0.01	2.51 ± 0.02	3.52 ± 0.28	2.34 ± 0.01
C_16:0 (Palmitic)_	13.36 ± 0.05	17.44 ± 0.05	24.67 ± 0.66	15.13 ± 0.03	12.76 ± 0.23	27.19 ± 0.17	17.38 ± 0.17	16.01 ± 0.02	15.82 ± 0.20	17.79 ± 0.07	19.27 ± 0.03	13.68 ± 1.22	14.43 ± 0.07	16.54 ± 0.02	34.17 ± 0.22	17.61 ± 0.04
C_16:1 (Palmitoleic)_	nd	nd	nd	nd	0.74 ± 0.15	nd	nd	nd	nd	nd	nd	nd	nd	nd	nd	nd
C_18:0 (Stearic)_	1.27 ± 0.32	2.42 ± 0.09	10.03 ± 2.07	1.55 ± 0.36	1.35 ± 0.20	2.22 ± 0.59	3.21 ± 0.63	1.54 ± 0.03	1.51 ± 0.10	1.84 ± 0.13	6.01 ± 0.12	1.18 ± 0.54	2.01 ± 0.01	1.76 ± 0.10	6.24 ± 0.60	1.82 ± 0.44
C_18:1 (Oleic)_	17.90 ± 0.11	20.51 ± 0.02	24.95 ± 0.60	19.51 ± 0.08	9.16 ± 0.26	9.20 ± 0.07	9.12 ± 0.07	10.18 ± 0.06	14.09 ± 0.05	21.68 ± 0.08	15.74 ± 0.04	18.08 ± 1.11	3.43 ± 0.03	3.64 ± 0.03	10.99 ± 0.60	3.86 ± 0.02
C_18:2 (C9, t11)_	34.02 ± 0.19	30.68 ± 0.02	17.07 ± 0.28	32.38 ± 0.13	34.72 ± 0.30	23.12 ± 0.13	29.57 ± 0.13	32.46 ± 0.08	31.52 ± 0.03	31.17 ± 0.11	29.08 ± 0.05	30.17 ± 1.71	8.53 ± 0.04	8.75 ± 0.05	nd	8.38 ± 0.03
C_18:2 (C9, 12)_	32.62 ± 0.18	28.96 ± 0.04	23.28 ± 0.53	31.00 ± 0.07	40.46 ± 0.33	35.57 ± 0.20	40.72 ± 0.26	39.10 ± 0.13	36.34 ± 0.06	26.37 ± 0.09	29.90 ± 0.04	36.14 ± 4.60	69.70 ± 0.13	66.80 ± 0.04	66.80 ± 0.04	66.00 ± 0.37
SFAs	15.47 ± 0.47	19.86 ± 0.04	34.70 ± 1.41	17.11 ± 0.27	14.92 ± 0.32	32.11 ± 0.40	20.59 ± 0.46	18.26 ± 0.26	18.06 ± 0.05	20.78 ± 0.28	25.28 ± 0.11	15.48 ± 0.44	18.35 ± 0.08	20.81 ± 0.11	43.94 ± 0.36	21.76 ± 0.41
MUFAs	17.90 ± 0.11	20.51 ± 0.02	24.95 ± 0.60	19.51 ± 0.08	9.89 ± 0.19	9.20 ± 0.07	9.12 ± 0.07	10.18 ± 0.06	14.09 ± 0.05	21.68 ± 0.08	15.74 ± 0.04	18.08 ± 1.11	3.43 ± 0.03	3.64 ± 0.03	10.99 ± 0.06	3.86 ± 0.02
PUFAs	66.64 ± 0.36	59.63 ± 0.05	40.35 ± 0.81	63.38 ± 0.19	75.18 ± 0.36	58.69 ± 0.33	70.29 ± 0.39	71.56 ± 0.21	67.85 ± 0.09	57.54 ± 0.20	58.98 ± 0.08	66.47 ± 0.19	75.22 ± 0.11	75.55 ± 0.08	45.08 ± 0.30	74.38 ± 0.40

Abbreviations: Glycer: Glycerides; Glyco: Glycolipids; Phospho: Phospholipids.

### 3.3 Composition of glycerides, glycolipids, and phospholipids

The exact lipid molecular compositions of the four mushroom oils were detected by UPLC/MS, 62 glycerides, 7 glycolipids, 7 phospholipids, and 3 sterols were identified, as shown in Table 3. The area normalization method was applied to quantify the different lipid components, and glycerides with more than 95% content, were thought to be the majority of four mushroom oils, which were found to represent a larger percentage of phospholipids than glycolipids.

**TABLE 3:** Lipid molecular composition of four test mushroom oils.

Content (%)/mean ± SD (*n* = 3)															
No	RT	MS2Metabolite	m/z	AC	LE	FM	VV	No	RT	MS2Metabolite	m/z	AC	LE	FM	VV
**(a) Glycerides compositions of four test mushroom oils (%)**															
	**Glycerides**			96.00 ± 4.07^a^	97.26 ± 4.86^a^	96.15 ± 2.83^a^	94.61 ± 2.07^a^								
	**TAG**			18.44 ± 1.03^a^	13.22 ± 0.53^c^	17.39 ± 1.81^ab^	16.52 ± 1.82^b^								
1	7.09	15:0_18:2_18:3	856.74	9.03 ± 0.26^bc^	23.00 ± 2.55^a^	10.44 ± 0.76^b^	8.76 ± 1.28^c^	12	7.99	18:1_18:1_19:2	914.81	nd	3.63 ± 0.67	nd	nd
2	7.32	15:1_16:0_18:2	832.73	1.94 ± 0.3^d^	5.60 ± 1.47^a^	2.41 ± 0.10^c^	3.71 ± 0.64^b^	13	8.01	16:0_18:2_18:2	872.77	4.61 ± 0.33^b^	2.12 ± 0.11^c^	nd	12.25 ± 0.58^a^
3	7.48	15:0_18:1_18:2	860.77	7.83 ± 0.49^b^	21.51 ± 1.26^a^	8.40 ± 0.65^b^	nd	14	8.07	18:1_18:2_21:2	940.83	6.48 ± 0.13^a^	nd	3.51 ± 0.19^b^	nd
4	7.51	15:1_16:0_18:1	834.75	2.07 ± 0.17^c^	6.79 ± 0.41^a^	2.51 ± 0.20^b^	nd	15	8.17	18:1_18:1_18:2	900.79	10.78 ± 0.94^a^	4.51 ± 0.07^c^	9.34 ± 0.34^a^	5.06 ± 0.38^b^
5	7.58	18:2_18:3_19:1	910.78	nd	2.64 ± 0.16	nd	nd	16	8.19	16:0_18:1_18:2	874.78	4.90 ± 0.30^a^	2.1 ± 0.37^c^	nd	3.18 ± 0.21^b^
6	7.63	18:2_18:2_18:3	894.75	nd	nd	nd	14.89 ± 0.76	17	8.25	18:1_18:2_21:1	942.84	5.96 ± 0.16	nd	nd	nd
7	7.65	16:0_17:1_18:1	862.78	3.65 ± 0.78^b^	7.27 ± 1.43^a^	nd	nd	18	8.35	16:0_18:1_20:2	902.81	7.81 ± 1.45^a^	2.61 ± 0.16^c^	nd	3.74 ± 0.18^b^
8	7.76	18:1_18:2_19:2	912.79	nd	5.93 ± 0.29	nd	nd	19	8.35	16:0_18:1_18:1	876.79	3.88 ± 0.27^b^	nd	7.39 ± 1.33^a^	2.92 ± 0.11^c^
9	7.79	16:1_18:2_20:3	896.77	12.44 ± 0.41^c^	5.54 ± 0.07^d^	15.76 ± 0.34^b^	34.58 ± 1.90^a^	20	8.62	18:0_18:2_30:5	1062.94	3.41 ± 0.38^a^	nd	3.52 ± 0.35^a^	nd
10	7.90	15:0_18:2_18:2	858.75	nd	nd	nd	2.6 ± 0.17	21	8.77	18:0_22:3_26:3	1064.95	2.12 ± 0.16^a^	nd	2.6 ± 0.21^a^	nd
11	7.98	18:1_18:2_18:2	898.78	13.09 ± 0.61^a^	6.71 ± 0.90^d^	12.12 ± 0.13^b^	8.3 ± 0.39^c^								
	**DAG**			8.93 ± 0.19^b^	7.64 ± 0.55^b^	8.32 ± 0.35^b^	17.59 ± 2.33^a^								
1	4.83	DG 15:1_18:2	594.51	30.08 ± 2.72^b^	46.55 ± 2.90^a^	42.68 ± 2.16^a^	42.05 ± 5.53^a^	6	6.41	DG 18:1_18:2	636.55	16.65 ± 0.50^a^	11.97 ± 0.79^c^	13.59 ± 0.30^b^	7.86 ± 1.27^d^
2	5.27	DG 15:1_18:1	596.52	12.67 ± 0.41^c^	20.39 ± 1.47^a^	15.44 ± 0.56^b^	4.52 ± 1.63^d^	7	6.55	DG 19:0_20:3	678.60	5.98 ± 0.16^a^	nd	4.42 ± 0.16^b^	nd
3	6.13	DG 18:2_18:2	634.54	8.03 ± 0.37^b^	5.12 ± 0.32^d^	6.60 ± 0.48^c^	31.41 ± 2.76^a^	8	6.69	DG 18:1_18:1	638.57	8.50 ± 0.46^a^	4.51 ± 0.18^b^	8.44 ± 0.11^a^	nd
4	6.28	DG 21:1_18:3	676.58	4.55 ± 0.10	nd	nd	nd	9	8.13	DG 18:2_21:2	676.60	10.30 ± 1.84^a^	11.50 ± 0.48^a^	8.84 ± 1.33^ab^	6.58 ± 0.56^b^
5	6.40	DG 16:0_18:2	610.54	3.23 ± 0.14^b^	nd	nd	7.55 ± 0.51^a^								
	**MAG**			0.39 ± 0.01^b^	0.50 ± 0.00^ab^	0.59 ± 0.00^a^	0.19 ± 0.02^c^								
1	3.08	MG 16:0	348.33	nd	13.75 ± 0.43	nd	nd	5	3.66	MG 22:3	426.36	36.13 ± 2.09^b^	17.33 ± 0.61^c^	31.27 ± 1.29^b^	48.57 ± 2.18^a^
2	3.27	MG 18:1	374.33	11.36 ± 1.21^a^	5.21 ± 0.39^b^	9.87 ± 0.55^a^	nd	6	3.83	MG 21:2	414.35	nd	nd	8.54 ± 0.52	nd
3	3.32	MG 16:0	348.33	nd	4.96 ± 0.96	nd	nd	7	4.27	MG 22:2	428.37	22.53 ± 1.46^a^	12.03 ± 0.4^b^	25.63 ± 0.29^a^	17.34 ± 3.94^ab^
4	3.65	MG 21:2	414.34	nd	nd	nd	18.52 ± 0.91	8	4.73	MG 24:2	456.40	29.93 ± 0.18^b^	46.75 ± 0.86^a^	24.51 ± 0.09^c^	15.55 ± 1.59^d^
	**FFA**			67.92 ± 3.23^ab^	64.93 ± 4.3^b^	70.26 ± 0.88^a^	63.73 ± 5.99^b^								
1	2.65	FA 18:3	277.22	2.82 ± 0.28^b^	8.31 ± 0.55^a^	1.96 ± 0.10^c^	9.15 ± 0.93^a^	6	3.53	FA 18:1	281.25	37.65 ± 0.48^a^	35.63 ± 1.31^a^	38.33 ± 0.85^a^	18.44 ± 1.25^b^
2	2.87	FA 16:1	253.22	1.88 ± 0.17^ab^	1.91 ± 0.12^a^	1.76 ± 0.09^b^	1.03 ± 0.27^c^	7	4.27	FA 18:0	283.26	14.98 ± 0.44^b^	7.94 ± 0.22^c^	16.71 ± 0.49^a^	2.56 ± 0.13^d^
3	2.97	FA 18:2	279.23	35.51 ± 0.72^c^	40.29 ± 1.39^b^	33.35 ± 0.73^c^	54.41 ± 1.75^a^	8	6.08	FA 24:0	367.35	1.78 ± 0.02^a^	1.27 ± 0.04^b^	1.79 ± 0.12^a^	2.03 ± 0.18^a^
4	3.15	FA 15:0	241.22	nd	nd	nd	1.23 ± 0.06	9	6.28	FA 25:0	381.37	nd	nd	nd	1.7 ± 0.31
5	3.46	FA 16:0	255.23	5.31 ± 0.62^b^	3.6 ± 0.06^c^	5.14 ± 0.49^b^	7.65 ± 0.33^a^	10	6.48	FA 26:0	395.38	nd	1.06 ± 0.09^b^	0.94 ± 0.06^c^	1.86 ± 0.32^a^
	**Ether Glycerides**			4.39 ± 0.11^b^	13.76 ± 0.29^a^	3.44 ± 0.13^c^	2.07 ± 0.09^d^								
1	6.12	O‐8:0_10:0_16:1	617.51	nd	nd	nd	15.53 ± 0.73	8	6.95	O‐19:2_17:2	620.56	7.83 ± 0.54^a^	3.04 ± 0.22^b^	7.46 ± 0.55^a^	7.53 ± 0.55a
2	6.52	O‐8:0_18:2_18:3	749.62	13.72 ± 0.12^b^	nd	15.83 ± 0.56^a^	nd	9	6.8	O‐19:2_18:3	632.56	15.15 ± 1.19^b^	31.47 ± 0.21^a^	9.63 ± 0.69^c^	27.51 ± 1.96^a^
3	7.04	O‐8:0_18:1_18:2	753.65	6.91 ± 0.34^b^	nd	8.55 ± 0.30^a^	nd	10	7.06	O‐19:2_18:2	634.57	22.29 ± 2.23^b^	33.92 ± 0.31^a^	18.05 ± 2.17^b^	nd
4	6.65	O‐19:2_16:3	604.53	nd	nd	nd	11.93 ± 0.61	11	7.34	O‐19:1_18:2	636.59	8.46 ± 0.68^b^	14.52 ± 0.07^a^	9.27 ± 1.34^b^	nd
5	6.8	O‐19:2_16:2	606.54	nd	1.8 ± 0.05	nd	nd	12	7.61	O‐19:1_18:1	638.6	nd	3.47 ± 0.12^a^	3.57 ± 0.25^a^	nd
6	7.08	O‐19:2_16:1	608.56	nd	6.22 ± 0.07	nd	nd	13	7.27	O‐22:6_21:2	710.61	25.86 ± 3.57^a^	2.12 ± 0.24^b^	27.42 ± 5.11^a^	22.25 ± 4.34^a^
7	7.35	O‐19:1_16:1	610.57	nd	3.4 ± 0.08	nd	nd	14	7.09	O‐22:6_22:3	722.6	nd	nd	nd	15.24 ± 1.09
**(b) Glycolipid, phospholipid, and sterol compositions of four mushroom lipids (%)**															
	**Glycolipids**			0.91 ± 0.06^b^	0.54 ± 0.03^c^	0.91 ± 0.08^b^	1.41 ± 0.06^a^								
1	2.39	MGDG O‐4:0_14:1	577.35	nd	nd	nd	1.69 ± 0.07	5	6.20	MGDG O‐13:0_15:0	683.50	nd	6.74 ± 0.28^c^	17.85 ± 2.47^a^	7.83 ± 1.05^b^
2	4.42	SMGDG O‐8:0_18:0	711.43	nd	7.75 ± 0.59^a^	nd	1.62 ± 0.20^b^	6	6.75	DGDG O‐8:0_22:1	871.57	15.93 ± 2.32^a^	nd	nd	nd
3	4.74	MGDG O‐9:0_18:2	701.45	nd	nd	82.02 ± 2.47^a^	84.63 ± 0.73^a^	7	7.70	DGDG O‐27:0_26:0	1195.94		nd	nd	1.98 ± 0.44^a^
4	6.06	MGDG O‐9:0_18:3	663.45	84.13 ± 2.30^a^	85.60 ± 0.87^a^	nd	2.20 ± 0.27^b^								
	**Phospholipids**			1.64 ± 0.23^bc^	1.88 ± 0.05^b^	2.01 ± 0.02^a^	1.40 ± 0.23^c^								
1	1.82	PE‐Cer 15:1;2O/14:1	587.42	20.21 ± 1.33^c^	24.86 ± 1.56^b^	25.02 ± 1.98^ab^	26.78 ± 3.77^a^	5	5.97	Cer‐NS 21:3;2O/18:2	600.53	nd	nd	15.9 ± 0.37^b^	38.82 ± 2.12^a^
2	3.33	SM 8:0;2O/20:4	613.44	13.37 ± 0.8^a^	nd	nd	11.82 ± 3.02^b^	6	6.06	Cer‐BS 19:1;2O/16:2;(3OH)	562.48	nd	nd	19.8 ± 0.55^a^	nd
3	4.06	PE‐Cer 16:2;2O/13:0	587.41	nd	nd	nd	9.09 ± 3.46	7	6.24	Cer‐BS 19:1;2O/16:2;(3OH)	562.48	66.44 ± 0.56^b^	75.12 ± 1.46^a^	39.31 ± 2.29^c^	nd
4	5.72	SM 44:3;3O	855.70	nd	nd	nd	13.4 ± 1.64								
	**Sterols ester**			1.58 ± 0.23^a^	0.36 ± 0.05^b^	1.52 ± 0.29^a^	1.57 ± 0.25^a^								
1	8.30	BRSE 28:2/18:2	678.61	60.47 ± 2.47^a^	60.38 ± 0.33^a^	58.94 ± 1.82^a^	34.07 ± 3.32^b^	3	8.62	CASE 28:1/18:1	682.64	4.64 ± 0.28^b^	5.86 ± 0.02^ab^	6.63 ± 1.78^a^	3.01 ± 0.41^c^
2	8.43	CASE 28:1/18:2	680.63	34.84 ± 2.26^b^	33.81 ± 0.37^b^	34.46 ± 0.53^b^	62.91 ± 3.73^a^								

*Note*: Datas with different superscript letter mean a significant difference (*p* < .05) betwwen two samples, while that with same letter signifys no significant difference.

Abbreviations: AC, *Agrocybe cylindracea*; FM, *Flower mushroom*; LE, conventional *Lentinula edodes*; VV, *Volvariella volvacea*.

#### 3.3.1 Analysis of glyceride composition

A total of 21 triglycerides (TAGs), 9 diglycerides (DAGs), 8 monoglycerides (MAGs), 10 free fatty acids (FFAs), and 14 ether‐glycerides were detected in the tested mushroom oils. But the shared lipid components were made up of 7 FFAs, 5 TAGs, 5 DAGs, 3 MAGs, and 3 ether‐DAGs. More FFAs (63.73–70.26%) were found than TAGs (13.22–18.44%), and linoleic acid (C18:2) at 33.35%–54.41% content was taken as the most important FFA of the four mushroom oils, conforming to the results above in Section 3.2 and a previous report (Gunc Ergonul et al., 2013; Kavishree et al., 2008). Similar to the FFAs distribution, the four mushroom oils also had the same dominant DAGs (i.e., C15:1/18:2) at 30.08–46.55%. Apart from FFA and DAG, the main ingredient of other glycerides section varied by mushroom species. For example, the TAG and ether‐DAG with the largest contents in *Volvariella volv*acea oil were C16:1/18:2/20:3 (34.58%) and C o‐19:2/18:3 (27.51%), respectively, while those in conventional *L. edodes* oil were C15:0/18:2/18:3 (23.00%) and C o‐19:2/18:2 (33.92%).

Overall, DAG content and ether‐glyceride (at 7.64–17.59% and 2.07–13.76% content) were found to be the most variable components of glyceride among the four oils. For example, the largest content of DAG (17.59%) but the lowest ether‐glyceride (2.07%) appeared in *Volvariella volvacea* glyceride, but the opposite situation was only shown in conventional *L. edodes* glyceride. As ether‐glyceride and DAG were mainly applied as cellular mediators (Snyder et al., 2002) and antiobesity substances (Lee et al., 2020), respectively, it was natural to assume that conventional *L. edodes* oil and *Volvariella volvacea* oil performed different functionalities, that is, cell signaling regulation and weight loss. Mushroom glycerides were first reported to contain ether‐glyceride at a content of 2.07–13.76% and conventional *L. edodes* oil was proved to be an excellent source of ether‐glyceride.

#### 3.3.2 Analysis of glycolipid, phospholipid, and sterol composition

Three minor lipids (≤2.10%) containing glycolipids, phospholipids, and sterols of the tested mushroom oils were analyzed in Table 3b, and the exact molecular compositions and contents were detected in different species. However, in any case, glyceryl phosphatide, cerebroside (Cer), and phytosterol esterified with linoleic acid (C18:2) were the main polar lipids of mushroom oils. Compared to other mushroom oils, *Volvariella volvacea* oil was discovered to have more diverse polar lipid molecules, that is, 6 glycolipids, 5 phospholipids, and 3 sterol esters. More phospholipids than glycolipids and sterol‐esters were found to exist in most mushroom oils except for *Volvariella volvacea* oil, which presented glycolipids, phospholipids, and sterol‐esters at similar proportions.

*Volvariella volvacea* oil contained Cer‐NS 21:3;2O/18:2 and CASE 28:1/18:2 as its main ingredients of phospholipids and sterols, which was different from other mushroom oils with the same main components of phospholipids and sterols, that is, Cer‐BS 19:1;2O/16:2;(3OH) and BRSE 28:2/18:2. However, this situation changed when discussing the glycolipid content in mushroom oils, as MGDG O‐9:0/18:3 was the main glycolipid of *Agrocybe cylindracea* and conventional *L. edodes* oils, while MGDG O‐9:0/18:2 was main glycolipid of *flower mushroom* and *Volvariella volvacea* oils. On the whole, *Volvariella volvacea* oil obviously had a higher content and molecular composition of polar lipids (5.39%, 14 molecules) than the other oils (*flower mushrooms*, approximately 4%, 9 molecules at most), consistent with the results described in Section 3.1.

### 3.4 Trace components

#### 3.4.1 Total phenol, flavone, and tocopherol contents

**FIGURE 1:**
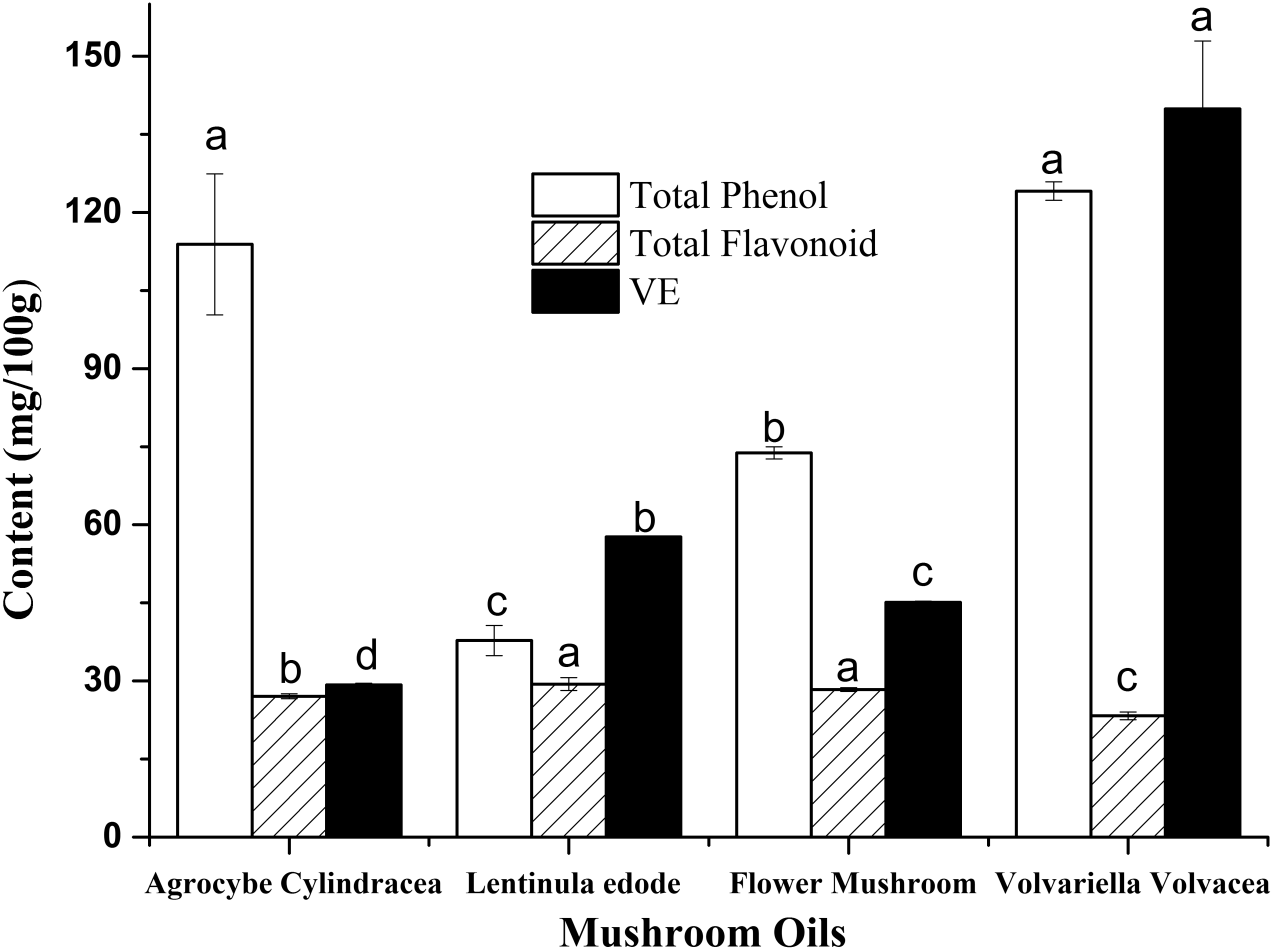
Content of total phenol, flavone and tocopherol in four mushroom oils.

Although phenolic compounds, flavones, tocopherols, and sterols were poorly soluble in oil, they are still regarded as effective antioxidants responsible for the storage stability of high‐PUFA oil. Tocopherol, a fat‐soluble vitamin, has often been studied owing to its antioxidant activity; however, in four tested mushroom oils, α‐tocopherol was identified as the only tocopherol analog. According to Figure 1, the four mushroom oils were discovered to have greater total phenol and tocopherol contents than flavone, which varied less from mushroom species. Distinguished from two mushroom oils (conventional *L. edodes* and *flower mushroom* oils) containing flavone at 29.390 mg/100 g and 28.34 mg/100 g, *Volvariella volvacea* oil appeared to have the lowest flavone content (23.28 mg/100 g) but the highest amount of total phenol (124.081 mg/100 g) and tocopherol (139.86 mg/100 g) among the four tested oils. Therefore, based on the large amount of antioxidants (total phenols and tocopherol), *Volvariella volvacea* oil would be bound to exhibit stronger free radical scavenging ability.

#### 3.4.2 Unsaponifiable matters

Oil unsaponifiable matters are some substances that cannot be saponified by strong basic solutions in oil, with the sterol component as the main component, oil unsaponifiable matter has often been studied, as sterol is often reported to present strong activities of lowering blood LDL‐cholesterol (Products and Allergies, 2014). GC–MS was applied to detect the unsaponifiable matter in four mushroom oils, which were quantified by internal standard 5α‐cholestane. According to the data in Table 4, 22 unsaponifiable matters containing 20 sterols and 2 nonsterols were identified in the four mushroom oils. The maximum amount of unsaponifiable matter existed in *Volvariella volvacea* oil (319.09 mg/kg), followed *by Agrocybe cylindracea* (183.59 mg/kg) and conventional *L. edodes* (190.60 mg/kg) oils. Egosterol and its analog were confirmed as the principal sterol components, as ergosterols in *Volvariella volvacea*, *conventional L. edodes*, *Agrocybe cylindracea*, and *flower mushroom* oils occupied 75.98%, 71.80%, 60.98%, and 56.26% of the unsaponifiable matter respectively, endowing *Volvariella volvacea* oil with optimal activity to improve osteoporosis (Morales et al., 2017). Moreover, stigmasterols and lanosterols were also found in four mushroom oils at amounts of 21.63–40.01 mg/kg and 2.70–7.56 mg/kg, and the existence of nonsterols such as lupeol and friedelan‐3‐one (7.85–29.67 mg/kg) benefited four tested oils with antimicrobial, anti‐inflammatory, and anticonvulsant activities (Gallo & Sarachine, 2009; Ichiko et al., 2016; Jordan et al., 2020; Sousa et al., 2017).

**TABLE 4:** Compositions of the unsaponifiable matter of four test mushroom oils (mg/kg).

Content (mg/kg)/mean ± SD (*n* = 3)													
No	RT	Name	AC	LE	FM	VV	No	RT	Name	AC	LE	FM	VV
		Ergosterol and its analog	111.95 ± 1.00^c^	136.86 ± 12.46^b^	48.08 ± 2.42^d^	242.45 ± 2.51^a^							
1	11.4	Ergosta‐5,7,9(11),22‐tetraen‐3‐ol, (3β,22E)‐	nd	9.85 ± 0.91^a^	0.93 ± 0.14^b^	9.29 ± 0.04^a^	6	14.85	5.Xi.‐Ergost‐7‐en‐3β‐ol, TMS derivative	26.01 ± 0.25^b^	36.60 ± 3.31^a^	nd	nd
2	13.44	Ergosterol	55.51 ± 0.43^b^	59.20 ± 5.46^b^	0.64 ± 0.00^c^	117.67 ± 1.04^a^	7	16.32	9,19‐Cyclolanost‐24‐en‐3‐ol,3β, TMS derivative	3.57 ± 0.06^a^	1.74 ± 0.02^b^	0.74 ± 0.16^c^	1.57 ± 0.09^b^
3	14.04	Ergosta‐7,22‐dien‐3‐ol, (3β,22E)‐, TMS derivative	13.91 ± 0.16^b^	12.51 ± 1.25^b^	13.85 ± 0.70^b^	21.59 ± 0.21^a^	8	17.51	9,19‐Cycloergost‐24(28)‐en‐3‐ol, 4,14‐dimethyl‐, acetate, (3β.,4α,5α)‐	2.50 ± 0.08^b^	nd	2.26 ± 0.48^b^	4.3 ± 0.03^a^
4	14.35	Ergost‐8(14)‐en‐3‐ol, 3β‐, TMS derivative	2.21 ± 0.06^c^	3.00 ± 0.29^c^	23.41 ± 1.23^b^	73.38 ± 0.59^a^	9	17.75	Ergost‐25‐ene‐3,5,6,12‐tetrol, (3β,5α,6β,12β)‐	nd	nd	0.13 ± 0.18^b^	3.57 ± 0.24^a^
5	14.58	Ergosta‐5,8‐dien‐3‐ol, 3β‐	8.24 ± 0.10^ab^	9.27 ± 0.93^ab^	6.12 ± 0.24^b^	11.08 ± 2.89^a^							
		Stigmasterol and its analog	40.01 ± 0.36^a^	22.94 ± 2.24^b^	21.63 ± 1.05^b^	37.27 ± 0.07^a^							
1	13.35	Stigmasterol, TMS derivative	23.77 ± 0.24^a^	nd	14.41 ± 0.80^b^	6.67 ± 0.14^c^	3	15.15	Stigmast‐5‐ene, 3β‐(trimethylsiloxy)‐, (24S)‐	8.99 ± 0.08^b^	15.01 ± 1.50^a^	7.21 ± 0.33^c^	13.71 ± 0.12^a^
2	14.74	Silane, trimethyl[[3β‐stigmasta‐7,24(28)‐dien‐3‐yl]oxy]‐	7.26 ± 0.05^b^	7.93 ± 0.85^b^	nd	16.89 ± 0.00^a^							
		Lanosterol and its analog	4.35 ± 0.10^b^	7.56 ± 0.47^a^	2.70 ± 0.23^c^	4.81 ± 0.08^b^							
1	15.91	Lanosterol, TMS derivative	1.08 ± 0.09^b^	3.8 ± 0.38^a^	nd	nd	2	16.67	3β‐Lanosta‐7,9(11),24‐trien‐3‐ol, TMS derivative	3.27 ± 0.05^c^	3.76 ± 0.32^b^	2.70 ± 0.23^d^	4.81 ± 0.08^a^
		Other sterols	19.43 ± 0.19^a^	7.79 ± 1.34^b^	4.50 ± 0.32^c^	4.89 ± 0.10^c^							
1	13.63	Desmosterol, TMS derivative	2.23 ± 0.04	nd	nd	nd	4	15.34	Isofucosterol, O‐TMS	7.99 ± 0.05^a^	4.84 ± 1.14^b^	nd	nd
2	14.26	4‐Methylcholesta‐8,24‐dien‐3‐ol, (3β,4α,5α)‐, TMS derivative	3.16 ± 0.07^b^	2.95 ± 0.30^b^	2.20 ± 0.09^c^	4.89 ± 0.10^a^	5	15.33	Methyl (25RS)‐3α‐hydroxy‐5β‐cholestan‐26‐oate, TMS derivative	2.55 ± 0.02	nd	nd	nd
3	15.03	β‐Sitosterol, TMS derivative	3.50 ± 0.02	nd	nd	nd	6	17.17	Methyl 3‐hydroxycholest‐5‐en‐26‐oate, TMS derivative	nd	nd	2.30 ± 0.23	nd
		Non sterol	7.85 ± 0.12^c^	15.44 ± 0.91^b^	8.55 ± 0.52^c^	29.67 ± 0.26^a^							
1	16.22	Lupeol trimethylsilyl ether	4.64 ± 0.05^d^	8.87 ± 0.34^b^	5.31 ± 0.30^c^	12.56 ± 0.09^a^	2	18.65	Friedelan‐3‐one	3.21 ± 0.07^d^	6.57 ± 0.58^b^	3.24 ± 0.22^d^	17.11 ± 0.19^a^
		Total content	183.59 ± 1.74^b^	190.60 ± 17.52^b^	85.46 ± 4.37^c^	319.09 ± 2.62^a^							

*Note*: Datas with different superscript letter mean a significant difference (*p* < .05) betwwen two samples, while that with same letter signifys no significant difference.

Abbreviations: AC, *Agrocybe cylindracea*; FM, *Flower mushroom*; LE, conventional *Lentinula edodes*; VV, *Volvariella volvacea*.

### 3.5 Antioxidant activity evaluation

**FIGURE 2:**
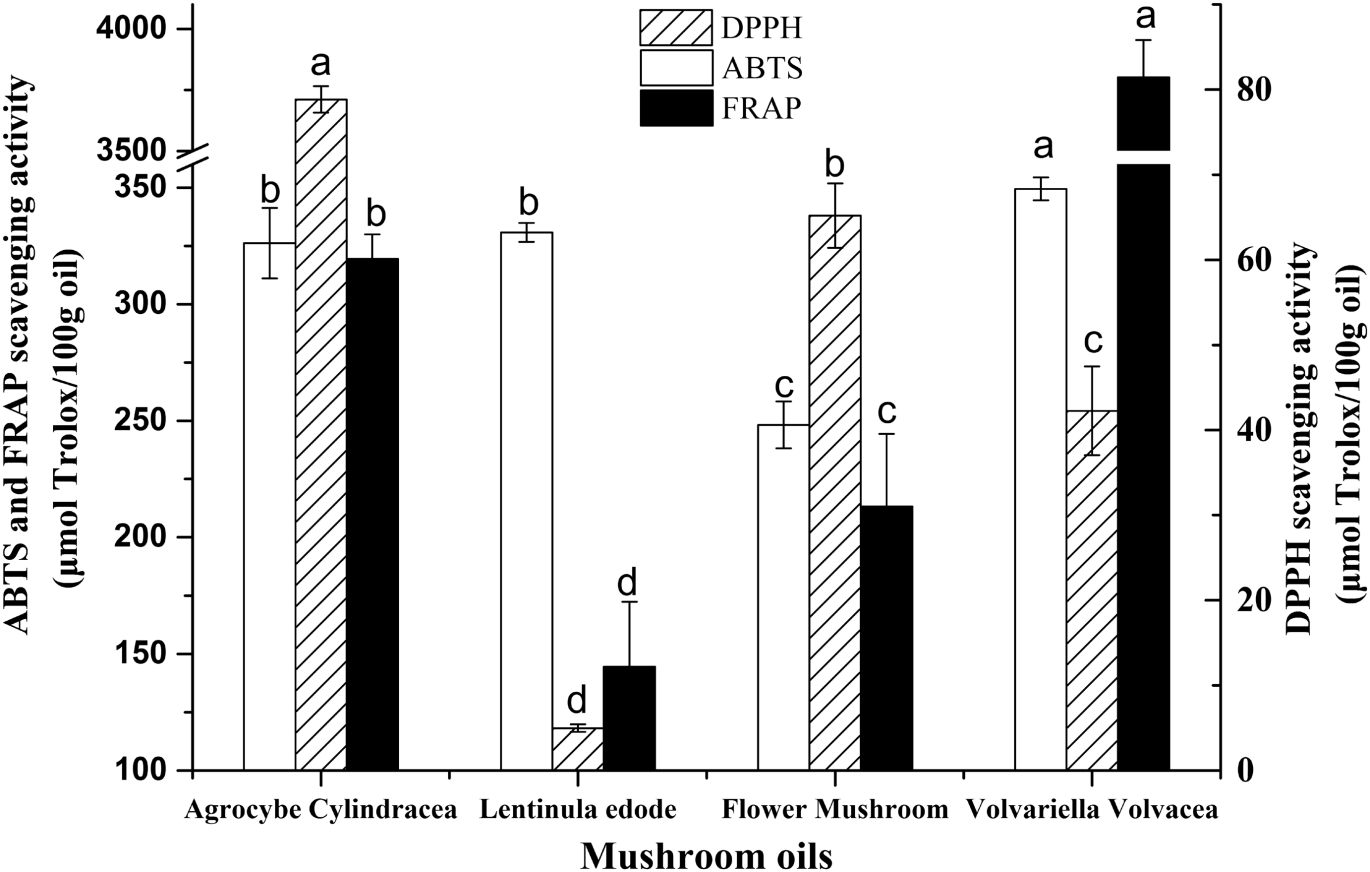
The antiradical scavenging activity of four mushroom oils.

The free radical scavenging capacity of the four mushroom oils was assessed by three indicators (i.e., values of DPPH, ABTS, and FRAP). According to Figure 2, the four tested oils all showed superior ABTS and FARP values (144.54–3801.70 μmol Trolox/100 g) superior to those of DPPH (4.99–78.84 μmol Trolox/100 g). Relative to the best DPPH value (78.84 μmol Trolox/100 g) in *Agrocybe cylindracea* oil, both the maximal ABTS (349.45 μmol Trolox/100 g) and FRAP (3801.70 μmol Trolox/100 g) values only appeared at *Volvariella volvacea* oil. The antioxidant activities of two *L. edodes* oils were not significant, as *flower mushroom* oil had DPPH, ABTS, and FRAP values at 65.18, 248.23, and 213.22 μmol Trolox/100 g, respectively, while conventional *L. edodes* oil presented poor DPPH and FRAP values. Overall, *Volvariella volvacea* oil was considered to have the optimal antioxidant activity, which mostly originated from its large amount of unsaponifiable substances, total phenols, and tocopherols.

## 4 CONCLUSION

Mushroom oil, acting as a nontraditional oil with abundant polar lipids and lipid accompaniment, was shown to display biological activities such as lowering cholesterol levels, and antibacterial, antioxidant, anti‐inflammatory, and anticonvulsant activities. In this paper, the optimal mushroom oil was selected by investigating the nutritional compositions and antioxidant activities of four mushroom oils. Based on superior indicators containing PUFAs, polar lipids, total phenols, α‐tocopherol, as well as ABTS and FRAP values, *Volvariella volvacea* oil was deemed the best oil among the four tested mushroom oils. Further investigations on related mushroom oils should focus on validating their biological activities as well as their development and application.

## AUTHOR CONTRIBUTIONS

**Jingjing Kang:** Conceptualization (equal); investigation (equal); resources (equal); validation (equal); writing – original draft (equal). **Yue Yue:** Formal analysis (equal); writing – review and editing (equal). **Shaofeng Wei:** Supervision (equal). **Huifang Chen:** Writing – review and editing (equal). **Peng Luo:** Project administration (equal).

## Data Availability

The data that support the findings of this study are available from the first author upon reasonable request.
